# The influence of the gingival phenotype on implant survival rate and clinical parameters: a systematic review

**DOI:** 10.1038/s41432-025-01114-x

**Published:** 2025-02-05

**Authors:** Diego Marques da Silva, Filipe Castro, Bruno Martins, Javier Flores Fraile, Juliana Campos Hasse Fernandes, Gustavo Vicentis Oliveira Fernandes

**Affiliations:** 1 FP-I3ID, Faculty of Health Science, Fernando Pessoa University, Porto, Portugal; 2 Doctoral Program “Surgery and Dentistry” Department of Surgery, University of Salamanca, Salamanca, Spain; 3 DDS, Private Researcher, St. Louis, MO USA; 4 Missouri School of Dentistry & Oral Health, A. T. Still University, St. Louis, MO USA

**Keywords:** Dental implants, Dental diseases

## Abstract

**Objective:**

The goal of this systematic review was to verify whether the gingival phenotype (thick or thin) could impact the dental implant survival rate by affecting the marginal bone.

**Methods:**

The search was carried out on PubMed/MedLine, PubMed Central, and B-On databases. The research question was: “Does gingival phenotype positively or negatively influence marginal bone loss around dental implants?” The inclusion criteria were: any clinical trial/study, comparative study, prospective or retrospective articles, systematic review that addressed at least a 1-year follow-up with an assessment of the marginal bone loss (MBL) around dental implants, articles that reported the gingival phenotype (thin or thick) and were published in the last 13 years. The exclusion criteria were narrative or other reviews, letters to the editor, and commentaries. Data extraction included the author’s name, year of publication, type of study, sample size, number of implants, method used, and outcomes presented. The extracted data was summarized and presented in the results section. Critical Appraisal tool in JBI Systematic Reviews was used to determine the possibility of bias.

**Results:**

A total of 62 articles were found, but eight articles were relevant to compose this study. After deep evaluation, it was possible to observe the implant success rate for both gingival phenotypes, thin and thick, was greater than 91% within a follow-up of up to 5 years. Therefore, it is unclear whether the thickness of the gingival tissue surrounding the implant can directly influence the marginal bone level. The gingival phenotype may be indirectly involved in the survival rate of dental implants, as it can be a risk factor for peri-implantitis, leading to marginal bone loss beyond what is expected.

**Discussion:**

The thin gingival phenotype is one of the main risk factors for additional bone loss. It is crucial to know how to preserve the healthy condition.

**Conclusion:**

Within the results found, the gingival phenotype is indirectly related to implant survival rate and clinical parameters, which were respectively high and non-conclusive. Therefore, a higher risk of peri-implantitis is suggested when a thin phenotype is present.

Key points

- A high implant success rate can be found for both gingival phenotypes (thin and thick), greater than 91% within a follow-up of up to 5 years.
- A flapless protocol can provide a better esthetic result in the short term.
- The thin gingival phenotype is one of the main risk factors for additional bone loss.

## Introduction

The pursuit of replacing missing teeth with dental implants has become a standard option in oral rehabilitation cases^1^. Increased comfort, biomimicry to the natural tooth, and functional restoration^2^, combined with a high implant survival rate^3,4^, allow implantology to present itself as a valid, safe, and predictable option in its practice in contemporary dentistry^5^.

The angle of the implant placement, platform, distance from the contact point of the prosthetic crown to the bone crest, bone crest height, implant placement procedure, placement time after extraction, peri-implant gingival anatomy, and phenotype are factors that directly influence the quality and stability of the peri-implant soft tissue^6,7^. Among these factors, gingival thickness has a direct influence not only on the esthetic outcome but also on long-term implant survival^8^. A thin gingival phenotype is associated with an increased risk of unfavorable outcomes after surgical procedures. Following the tooth extraction, significant morphologic and histologic changes occur in the alveolar crest, and the placement of juxta- or infra-osseous dental implants can cause vertical bone resorption, considering that the peri-implant mucosa requires a larger dimension than that of a natural tooth, and natural bone resorption occurs when the supracrestal tissue attachment (STA), former biological width, is reduced^9^.

It is believed that the mucosa, in its healthy state, provides a protective biological barrier for the functioning dental implant. However, implant survival can be reduced when they are affected by an inflammatory process (peri-implantitis), which is caused by infection around osseointegrated implants, resulting in marginal bone loss (≥2 mm) and formation of a peri-implant pocket due to intraoral migration of pathogenic microbial agents from adjacent teeth to the implant site^10^. Peri-implantitis is very similar to periodontitis, not only because it contains a microbiota with a prevalence of gram-negative anaerobic microorganisms but also because it presents clinical signs such as bleeding on probing and suppuration^11,12^.

Nagaraj et al.^13^ presented the characteristics of the thin periodontal phenotype as a soft, thin, delicate gingival tissue with minimal attached gingiva associated with thin and irregular underlying bone architecture characterized by bone dehiscence and fenestration. It responds to disease with a recession of the gingival margin. Its transparency can be a negative factor for esthetics, making metal substructures visible. On the other hand, the thick periodontal phenotype has been described as a thick, dense, and fibrous soft tissue with a large amount of attached gingiva and a relatively thick and flat underlying bone architecture.

The initial thickness of the mucosal tissue may be an essential factor in the etiology of peri-implant bone loss over time, suggesting that the gingival phenotype can be considered a risk factor in maintaining or promoting soft tissue integrity and maintaining marginal bone levels around the implant. Thus, this systematic review aimed to evaluate the influence of gingival phenotype (thin and thick) on dental implant survival.

## Materials and methods

This study followed the PRISMA (Preferred Reporting Items for Systematic Reviews and Meta-Analysis) guidelines. A bibliographic review was carried out based on scientific articles that study the influence of gingival phenotype on dental implant survival and clinical parameters at PubMed/MEDLINE, PubMed Central, and B-On (Online Library) databases. The research question was developed based on the PICO (Participants, Intervention, Comparison, and Outcomes) strategy: “Does gingival phenotype positively or negatively influence marginal bone loss around dental implants?” The research was conducted between April and September 2023. Based on the keywords: “Dental implants,” “survival,” “biotype,” “phenotype,” “periimplantitis,” and “peri-implant*,” seeking articles in the English language. The terms were combined using the Boolean markers “AND” and “OR”. Detailing the search strategy: ((“dental implants” OR “implant dentistry” OR “endosseous implants”) AND (“survival rates” OR “success” OR “longevity”)) AND ((“biotype” OR “tissue biotype” OR “soft tissue biotype”) AND (“phenotype” OR “phenotypic characteristics”)) AND ((“peri-implantitis” OR “peri-implant disease” OR “peri-implant inflammation”) AND (“peri-implant*“ OR “peri-implant health” OR “peri-implant tissue”)).

### Eligibility criteria

Two investigators (D.M.S. and F.C.) independently followed the search and selection of studies, and a third author (G.V.O.F.) collaborated in case of disagreement. The inclusion criteria were: (1) any clinical trial/study, (2) comparative study, (3) prospective or retrospective articles, (4) a systematic review that addressed at least a 1-year follow-up with an assessment of the marginal bone loss (MBL) around dental implants, (5) articles that reported the gingival phenotype (thin or thick), and (6) were published in the last 13 years. The exclusion criteria were narrative or other reviews, letters to the editor, and commentaries.

### Selection of studies and data extraction

Two authors (D.M.S. and F.C.) independently gathered all relevant information from the studies. A third author (G.V.O.F.) was consulted during any disagreements, which were resolved through a video meeting among the authors. Each author entered the necessary data into an Excel spreadsheet, which was later combined. The review process occurred in two stages: first, the titles and abstracts were examined, followed by the collection and assessment of the full articles based on the review criteria. References were screened for duplicates, which were eliminated. Subsequently, the titles and abstracts were assessed against predefined inclusion and exclusion criteria to identify potentially suitable studies. Full texts of eligible studies were read to confirm their appropriateness for inclusion. During the screening phase, secondary articles and those lacking comparison groups or baseline versus post-intervention data were also excluded.

Data extraction from the selected articles focused on the author’s name, publication year, study type, sample size, number of implants, methods employed, and reported outcomes. The summarized data was then presented in the results section.

### Quality assessment and risk of bias

Two independent investigators (J.C.H.F. and G.V.O.F.) performed the quality assessment, and in the case of divergences, a third researcher was consulted (F.C.). Critical Appraisal tool in JBI was used^14^, following the type of study included, to determine the possibility of bias. For systematic review, 11 questions filled out the form: “(1) Is the review question clearly and explicitly stated?; (2) Were the inclusion criteria appropriate for the review question?; (3) Was the search strategy appropriate?; (4) Were the sources and resources used to search for studies adequate?; (5) Were the criteria for appraising studies appropriate?; (6) Was critical appraisal conducted by two or more reviewers independently?; (7) Were there methods to minimize errors in data extraction?; (8) Were the methods used to combine studies appropriate?; (9) Was the likelihood of publication bias assessed?; (10) Were recommendations for policy and/or practice supported by the reported data?; and (11) Were the specific directives for new research appropriate?”.

For cohort studies, also 11 questions were raised: “(1) Were the two groups similar and recruited from the same population?; (2) Were the exposures measured similarly to assign people to both exposed and unexposed groups?; (3) Was the exposure measured in a valid and reliable way?; (4) Were confounding factors identified?; (5) Were strategies to deal with confounding factors stated?; (6) Were the groups/participants free of the outcome at the start of the study (or at the moment of exposure)?; (7) Were the outcomes measured in a valid and reliable way?; (8) Was the follow up time reported and sufficient to be long enough for outcomes to occur?; (9) Was follow up complete, and if not, were the reasons to loss to follow up described and explored?; (10) Were strategies to address incomplete follow up utilized?; and (11) Was appropriate statistical analysis used?”.

For cross sectional study, 8 items were assessed: “(1) Were the criteria for inclusion in the sample clearly defined?; (2) Were the study subjects and the setting described in detail?; (3) Was the exposure measured in a valid and reliable way?; (4) Were objective, standard criteria used for measurement of the condition?; (5) Were confounding factors identified?; (6) Were strategies to deal with confounding factors stated?; (7) Were the outcomes measured in a valid and reliable way?; and (8) Was appropriate statistical analysis used?”.

For case series, 10 questions were evaluated: “(1) Were there clear criteria for inclusion in the case series?; (2) Was the condition measured in a standard, reliable way for all participants included in the case series?; (3) Were valid methods used for identification of the condition for all participants included in the case series?; (4) Did the case series have consecutive inclusion of participants?; (5) Did the case series have complete inclusion of participants?; (6) Was there clear reporting of the demographics of the participants in the study?; (7) Was there clear reporting of clinical information of the participants?; (8) Were the outcomes or follow up results of cases clearly reported?; (9) Was there clear reporting of the presenting site(s)/clinic(s) demographic information?; and (10) Was statistical analysis appropriate?”.

Then, the risk of bias was sorted as: *low risk of bias* (plausible bias unlikely to alter the results seriously) if all criteria were met (all green [yes]) or at maximum two “unclear” were present; *moderate risk of bias* (“plausible bias” data raises some doubt about the results) if two “no” (red) was found or up to 4 “unclear” criteria were met; and *high risk of bias* (plausible bias that seriously weakens confidence in the results), at least 3 “no” (red) or ≥ 5 “unclear” was found.

## Results

**Fig. 1:**
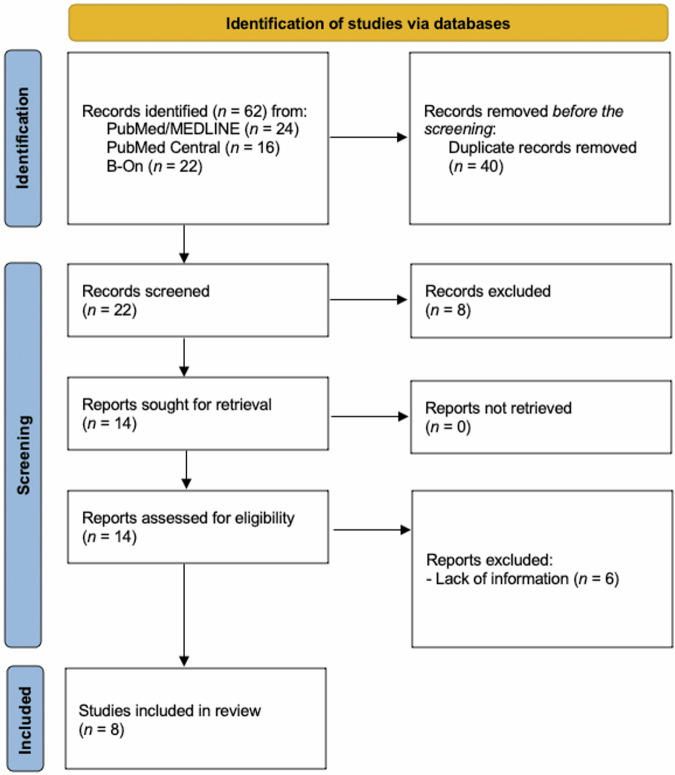
Flowchart for articles screening and selection.

A total of 62 articles were found; 40 duplicated articles were removed. After the title and abstract reading, 8 more articles were removed; 14 had the full text evaluated. Six articles were excluded due to the lack of adequate information. Thus, 8 articles^5,15–21^ met the eligibility criteria and were included in this research (Fig. 1). A total of 1417 implants and tissues were assessed. All the articles were detailed and summarized for a better understanding (Table 1). A full description, avoiding repetition, can be found below. In addition, after evaluating the articles included, a high level of heterogeneity was observed; this fact did not permit the development of a meta-analysis.

**Table 1:** Summarization of the included studies.

Study	Objective	Participants	Key Findings	Gingival phenotype findings
Cosyn et al.^14^	Evaluate immediate implants in the anterior maxilla after 3 years	30 patients	96% implant survival; significant increase in MBL (1.0 mm); probing depth decreased; esthetic results: 21% failures, 21% perfect, 58% acceptable.	Thick phenotype: significant mesial papilla growth.
Aguirre-Zorzano et al.^15^	Assess MBL around implants after one year, influence of periodontal therapy	49 patients	99.59% survival; MBL: 0.39 mm (SPT: 0.16 mm, no SPT: 0.62 mm); significant relation between MBL and plaque index/phenotype; higher peri-implantitis in thin phenotype.	Thin phenotype associated with increased bone loss and higher peri-implantitis incidence.
Guarnieri et al.^16^	Evaluate immediate implants with laser collar in maxilla	46 patients	95.6% survival; MBL increased to 0.58 mm at 2 years; gingival margin stable; 68% successful esthetic results.	Thick phenotype: stable gingival margins over time.
Stiller et al.^17^	Assess soft tissue grafting for peri-implantitis	28 patients	Significant KM increase (0.4 to 4.3 mm); probing depth decreased; all patients reported symptom improvement; unclear effect of phenotype on outcomes.	Thin phenotype more prevalent; phenotype impact on graft outcomes unclears.
Guarnieri et al.^18^	Compare MBL and esthetics after IIT and DIT	25 patients	100% survival; no significant MBL difference (IIT: 0.35 mm, DIT: 0.42 mm); slight papilla growth after IIT; esthetic results similar between groups.	Thick phenotype in both groups; slight papilla growth trend in IIT.
Weigl and Strangio ^19^	Systematic review on hard/soft tissue impacts on implants	17 studies	97.96% success rate; MBL < 1 mm; thick phenotype more prevalent; mucosal phenotype affected mid-facial levels but not MBL or papilla regeneration.	Thick phenotype found in 60.5% of sites; thin phenotype correlated with more mid-facial recession.
Guarnieri et al.^20^	Evaluate mucositis/peri-implantitis incidence based on implant surface	74 patients	Higher mucositis (22.8%) and peri-implantitis (7.8%) in nML group; MBL significantly lower in ML group (1.45 mm vs. 2.55 mm); significant differences in disease incidence by prosthesis type.	No significant differences in mucositis/peri-implantitis incidence based on phenotype.
Isler et al.^5^	Analyze clinical parameters and severity of peri-implantitis by phenotype	87 patients	25.3% smokers; thick phenotype had lower MBL; non-cooperation with maintenance increased disease severity risk.	Thick phenotype associated with lower MBL; thin phenotype linked to higher disease severity.

*MBL* marginal bone loss, *SPT* periodontal support therapy, *KM* keratinized tissue, *IIT* immediate implant treatment, *DIT* delayed implant treatment, *nML* non-laser engraved, *ML* laser engraved.

Cosyn et al.^15^ This article aimed to evaluate the results of treatments with immediate implants in the anterior region of the upper jaw after three years of function. They selected 30 patients who needed an implant in the esthetic zone of the maxilla with a thick gingival phenotype. Patients underwent a surgical process, which was carried out delicately by performing an atraumatic extraction to preserve the bone walls, paying particular attention to three-dimensional (3D) positioning during implant placement, followed by filling the gap with biomaterial and making a screw-retained provisional crown that was replaced after 6 months by a cement-retained definitive crown.

Three years after definitive loading, the patients were assessed clinically and radiographically to determine implant survival rate, complications, and the final condition of the hard and soft tissues. The esthetic result was objectively assessed using the pink esthetic score (PES) and the white esthetic score (WES). Only 25 patients were re-evaluated. The implant survival rate was 96%. After radiographic analysis, they observed that MBL increased significantly during the study period (*p* ≤ 0.038), with a mean MBL for the mesial and distal faces of 1.13 and 0.86 mm, respectively. Therefore, the overall average MBL was 1.0 mm. There was a significant reduction in probing depth from 3.46 to 3.17 mm (*p* = 0.015), contributing to a significant decrease in bleeding on probing (BoP) from 41 to 24% (*p* = 0.002). The mean mesial, distal, and buccal peri-implant tissue recession in relation to the preoperative state represented 0.05, 0.08, and 0.34 mm, respectively. The mesial papilla showed significant growth between the 1- and 3-year reassessments (0.36 mm; *p* = 0.015). 21% of the cases were considered esthetic failures, 21% showed an (almost) perfect result, and 58% showed acceptable esthetics.

Aguirre-Zorzano et al*.* ^16^ This retrospective observational study aimed to assess the MBL around implants after one year of function. The implants were placed in patients with a history of treated chronic periodontitis who underwent periodontal support therapy (SPT) or not. In addition, the influence of periodontal phenotype and plaque index on implant survival was also evaluated. A total of 49 patients took part in this study, 27 of whom underwent periodontal supportive therapy (SPT), while 22 willingly chose not to undergo SPT, even after the importance of the procedure and follow-up had been explained. The plaque index and gingival phenotype were assessed for each patient, and standardized radiographs were taken at the time of loading and one year later.

A total of 246 implants (Astra Tech® Osseo-Speed^TM^) were enrolled, 123 placed in patients with SPT and 123 without SPT. The survival rate was 99.59% due to the loss of one implant during the follow-up period in the non-SPT group. Six patients (12%) had peri-implantitis (1 in the SPT group and 5 in the no SPT group) and 16 (32%) mucositis (5 in the SPT group and 11 in the no SPT group). The average plaque index was 39.99% (20.34% in the SPT group and 59.63% in the no SPT group). The average MBL was 0.39 mm (0.16 mm in the SPT group and 0.62 mm in the no SPT group), with 118 SPT implants showing a lower-than-average loss in contrast to 66 implants in the no SPT group. A statistically significant relationship was demonstrated between bone loss around the implant and the patient’s periodontal phenotype and plaque index. Five of the 6 cases of peri-implantitis were related to a thin phenotype and only one to a thick biotype, while of the patients with mucositis, 12 were associated with a thin biotype and 4 to a thick biotype. The thin biotype represented a risk factor for additional bone loss.

Guarnieri et al.^17^ The aim was to evaluate the clinical, radiographical, and esthetical results of immediate implants with a micro-textured laser collar placed in the anterior region of the maxilla and restored with non-functional loading. 46 patients (24 men and 22 women) met the inclusion criteria: whose compromised tooth needed 4 mm of bone beyond the root apex, at least 10 mm of height, 4.5 mm of width bone available, absence of active periapical/periodontal lesions with a thick gingival phenotype, ideal gingival level/contour, and intact walls after extraction of the compromised tooth element. The implants were placed without a flap (flapless). Special attention was given to the 3D positioning and occlusal adjustment of the provisional crown so that it remained in infra-occlusion during the osseointegration period. The patients were followed up, and the plaque index, probing depth (PD), BoP, PES, and WES were evaluated at 6, 12, and 24 months.

The survival rate was 95.6%, as 2 implants were lost in the first 6 months. The average MBL (mesial and distal) was 0.41 mm (0.10–0.97 mm) and 0.47 mm (0.08–0.85 mm) at 6 months, 0.51 mm (0.17–1.05 mm) and 0.53 mm (0.32–1.00 mm) at 1 year, and 0.58 mm (0.17–1.15 mm) and 0.57 (0.42–1.10 mm) at 2 years, respectively. The gingival margin level showed no significant changes after 2 years. Loss of the mesial papilla was 1 mm in 1/44 (2.7%) of cases, and the distal papilla was 1 mm in 1/44 (2.7%) cases. In 36/44 (81.8%) and 34/44 (77.2%) patients, the mesial and distal papillae were at their original height, respectively. Regarding PES and WES, 68% of the cases were considered successful and 32% acceptable.

Stiller et al.^18^ This retrospective study evaluated soft tissue grafting as a surgical treatment option for peri-implantitis in the case of inadequate alveolar bone morphology and lack of keratinized mucosa. 28 patients (21 women, 7 men, mean age 59.4 years) suffering from peri-implantitis (with PD > 5 mm, with or without BoP, and with annual MBL > 0.2 mm) had agreed to take part in the investigation, in which they were treated with soft tissue grafting (STG).

The authors obtained a total of 54 implants that had been placed in the anterior/posterior region of the mandible and maxilla with keratinized oral mucosa ≤ 2 mm in height. Clinical investigations were carried out before surgery and after 9 to 180 months (average of 43 months), including the following clinical parameters: soft tissue phenotype, basic skeletal morphology of the alveolar bone, peri-implant keratinized mucosa (KM) width, PD, and BoP. Twenty-six patients had a thin phenotype, and 2 had a thick phenotype. Analysis of the basic skeletal morphology of the alveolar bone revealed a narrow apical base in 18 patients, a medium in 7 patients, and a wide base in 3 patients. KM width increased significantly (*p* < 0.01) from 0.4 ± 0.5 mm to 4.3 ± 1.5 mm after STG; PD was reduced considerably (*p* < 0.01) from 6.3 ± 2.3 mm to 4.1 ± 1.9 mm. A significant reduction (*p* < 0.01) in BoP was recorded. All patients reported a clinical improvement in inflammatory symptoms at follow-up. This study showed that STG can be successfully applied as a surgical treatment for peri-implantitis. However, it is unclear whether the soft tissues’ phenotype or the alveolar bone’s basic skeletal morphology affects the outcome of this surgical treatment.

Guarnieri et al.^19^ In this retrospective clinical study, the authors aimed to compare peri-implant MBL, soft tissue response, and esthetics after immediate implant treatment (IIT) and delayed implant treatment (DIT) in the esthetic zone of the maxilla using an implant with a laser-etched collar surface. They wanted to document the esthetic outcome of IIT and DIT as a secondary objective. Patients who required treatment with an implant in the anterior region of the upper jaw were selected for the study, provided they had mesial and distal teeth in the area where the implant would be placed, sufficient bone width to achieve primary implant stability, ideal soft tissue contour, and a thick gingival phenotype. The IIT group included immediate placement and provisionalization within 24 h of surgery, taking special care to ensure that there was no contact between the antagonist tooth and the provisional crown, while the DIT group included preservation of the extraction socket, followed by placement and provisionalization of the implant 4 months later.

A total of 25 patients were selected and divided into two groups according to the time of implant placement: 12 belonged to the IIT group and 13 to the DIT group. Each patient was checked every 6 months until the end of the 3 years. The level of the bone margin and the peri-implant condition of the mucosa were assessed at these regular intervals. The esthetic result was objectively evaluated after 3 years using the PES and WES. The authors found that the survival rate was 100% for both groups. The mean bone level ranged from 0.35 ± 0.18 mm for IIT to 0.42 ± 0.21 mm for DIT after 3 years (*p* > 0.05), with no difference between the groups. The mesial and distal papillae remained stable in the DIT group over time. A trend towards mesial and distal papillae growth was found after IIT (*p* < 0.05). The soft tissues of the mid-face remained stable over time after DIT and IIT, with a minimum average recession of 0.06 mm in IIT and 0.02 in DIT. The combined PES and WES results in the IIT group showed an almost perfect esthetic result in 4 out of 12 implants (33%); 6 out of 12 (50%) showed an acceptable result, and 2 out of 12 (17%) showed an unfavorable outcome. In the DIT group, 4 out of 13 (31%) had an almost perfect result, 8 out of 13 (61%) had an acceptable result, and 1 out of 13 (8%) had an unfavorable outcome.

Weigl and Strangio^20^. The authors conducted a systematic review that examined studies published between 2000 and 2015, focusing on the influence of hard and soft tissue on implant placement and immediate restoration. The review aimed to identify clinical parameters that impact outcomes. The authors evaluated various factors affecting hard and soft tissue results, including implant type, primary stability, gingival phenotype, flapless surgery, tooth extraction, implant arrangement, bone grafting techniques, the gap between the implant surface and the alveolar wall, and loading protocols.

The investigation included 17 studies, with a total of 411 implants placed without a flap and 215 with a mucoperiosteal flap. Notably, five studies mandated the elevation of the mucoperiosteal flap as part of the surgical protocol. The average “jumping space” between the implant surface and the alveolar wall was reported for 170 implants, measuring between 1.38 mm and 2.25 mm. Among the 201 implant sites that were not grafted, 405 received grafts, primarily using bone substitutes, though details were missing for 20 implants.

For 419 implants, a minimum insertion torque of ≥32 Ncm or an ISQ value of ≥ 60 was recorded, while 53 implants were accepted with a torque of 25 Ncm. Most implants were placed palatally within the mandibular bone, with the platform’s vertical position reported as 0.5 to 1.0 mm below the buccal bone crest or 3 to 4 mm apical to the adjacent cement-enamel junction (CEJ). A non-functional occlusion during post-insertion healing was observed in 97.8% of the cases, and definitive crowns were placed 3 to 6 months post-implantation. The review noted a success rate of 97.96% and a survival rate of 98.25% after an average follow-up period of 31.2 months.

The soft tissue phenotype was classified as thick in 379 sites (60.5%). Mean marginal bone loss (MBL) and interproximal mucosal changes were both less than 1 mm from baseline. The peri-implant mucosal level in the mid-facial area changed by less than 0.95 mm, regardless of whether the tissue was thin or thick, with no significant differences observed. While the mucosal phenotype negatively impacted mid-facial gingival levels, it did not affect MBL or papilla regeneration. Only one study in this systematic review indicated that the thin phenotype was associated with a significantly greater recession.

Guarnieri et al.^21^ In this retrospective study, the researchers aimed to evaluate the incidence of peri-implant mucositis and peri-implantitis around dental implants with the same body design but with a different bonding surface (laser engraved [ML] versus non-laser engraved [nML]) after 5 years in function. They selected 74 patients on a periodontal maintenance program every 3 or 6 months who received at least one implant with ML and one nML two-stage surgery. They were reopened 3 to 6 months after implant placement and crown fabrication and loading at 8/10 or 14/16 weeks. The following clinical variables were investigated: plaque index, PD, BoP, suppuration, and MBL around the implants and analyzed the correlation between the prevalence of mucositis/peri-implantitis and peri-implant phenotype (thick or thin), width of keratinized tissue (<2 mm or >2 mm), type of prosthetic connection (screwed or cemented), and type of prosthetic design (splintered or single).

In total, they obtained a sample of 166 implants, 82 of which were in the ML group and 84 in the nML group. In the ML group, 52 implants were splintered and 30 were not; 38 prostheses were cemented and 44 were screw-retained. In the nML group, 48 implants were splintered and 36 were not; 42 prostheses were cemented and 42 were screwed. 38 of the 166 implants had peri-implant mucositis (22.8%), which corresponds to 32.4% of patients, while 13 implants (7.8%) in 10 patients (13.5%) were diagnosed with peri-implantitis. 16 ML implants (19.5%) and 24 nML (28.5%) had peri-implant mucositis, while 3 of the ML group (3.6%) and 10 nML (11.9%) had peri-implantitis. The differences in the incidence of peri-implant disease between the groups were statistically significant (*p* < 0.05); for plaque index and the mean PD value of the implants in the ML group, no significant result was found, whereas, for the nML group, it was significant (*p* < 0.05), ranging from 4.4 ± 0.8 mm after 5 years.

Regarding BoP, no significant result was found for ML and nML, respectively: 84 out of 492 sites (17%) and 123 out of 504 sites (24.4%). For suppuration (P < 0.05): 14 out of 492 sites (2.8%) and 52 out of 504 sites (10.3%), respectively. Peri-implant PD ≥ 5 mm was found in 3 (3.6%) ML implants and 10 (11.9%) nML, while pockets ≥ 6 mm were found in 2 (2.4%) ML implants and 8 (9.5%) nML (*p* < 0.05). After 5 years of loading, the ML group had mean MBL values of 1.45 ± 0.21 mm and 1.57 ± 0.25 mm in the mesial and distal aspects, respectively. The nML group had mean values of 2.55 ± 0.25 mm and 2.61 ± 0.34 mm, respectively. The differences between the groups were statistically significant (*p* < 0.05). In both implant groups, there was a higher incidence of mucositis and peri-implantitis associated with cemented and splintered prostheses (*p* < 0.05), while the association with phenotype and keratinized mucosa width was not statistically significant (*p* > 0.05).

Isler et al.^5^ In this cross-sectional study, the authors analyzed clinical parameters (plaque index, PD, BoP, recession, clinical attachment loss (CAL), and MBL) and radiographic parameters in 7 different implant systems, which were analyzed and compared between the thin and thick phenotypes. Moreover, they assessed the severity levels of peri-implantitis. They identified possible risk indicators affecting the peri-implantitis severity, characterized by BoP and/or suppuration with ≥2 mm of MBL concerning the peri-implant gingival phenotype around dental implants.

A total of 87 patients participated in this investigation, with 229 implants diagnosed with peri-implantitis. Of these patients, 25.3% were current smokers, 59.7% had a history of periodontitis, and 32.1% currently had periodontitis. Based on the frequency of periodontal therapy maintenance visits, 9.3% of patients did not carry out maintenance, 50.5% carried out maintenance irregularly, and 40.2% cooperated with maintenance and attended appointments regularly.

The implants were divided into two groups according to the thickness of the surrounding mucosa: thin, ≤2 mm (TnB), and thick, >2 mm (TcB) thick. TcB and TnB were observed in 42.3% and 57.7% of implant sites, respectively. The distribution of peri-implantitis severity levels was 20.9% in SV1 (48/229), 19.2% in SV2 (44/229), 18.7% in SV3 (43/229), and 41.2% in SV4 (94/229), with SV1 being the simplest level and SV4 the most severe. The mean BoP, recession, CAL, and MBL values were significantly lower for the TcB group than the TnB group (*p* < 0.05). For plaque index and PD values, no significant differences were found between the groups according to the phenotype (*p* > 0.05). The authors also observed that implants with TnB in the group of patients who did not cooperate with periodontal maintenance had a significantly higher risk of progression of peri-implant disease to the highest degree of disease severity (from SV1 to SV4 level) compared to implants in the TnB group that regularly cooperated with periodontal maintenance therapy.

### Risk of bias

**Fig. 2:**
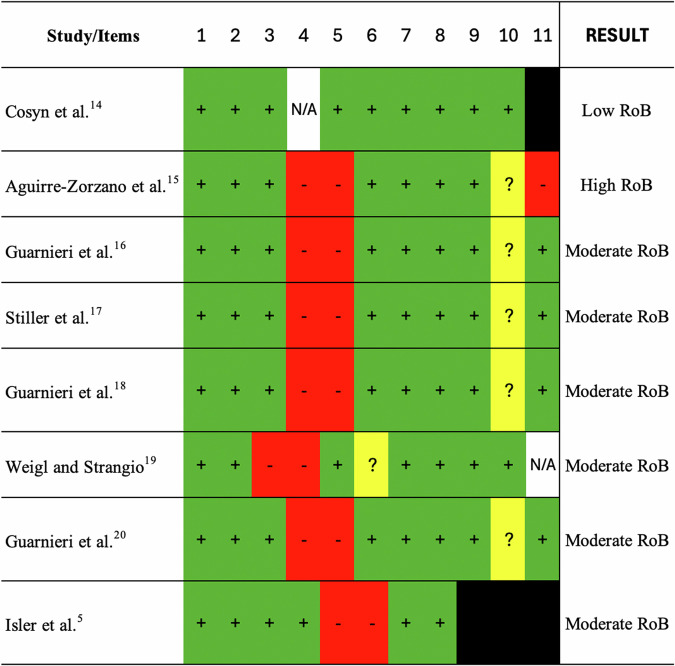
JBI system for evaluation of the Risk of Bias. (green +) = low risk; (yellow ?) = unclear; (red -) = high risk; N/A = not applied; black box = item not evaluated in the study.

All included studies were qualitatively assessed (Fig. 2). A low risk of bias was found in one study^15^; a high risk of bias was also observed in one study^16^; other six articles had a moderate risk of bias^5,17–21^.

## Discussion

The influence of gingival phenotype on dental implant survival has been a significant focus of research. Studies have shown that a thick gingival phenotype is associated with greater peri-implant mucosal thickness, which contributes to improving the resistance against mechanical stress and a lower incidence of gingival recession compared to thin phenotypes^22^. Specifically, the presence of thicker mucosa has been linked to reduced MBL and better esthetic results, as it minimizes the visibility of metal components through the gingival tissue^23^. Conversely, thin gingival tissues are more susceptible to recession and complications, such as midfacial peri-implant mucosal recession and incomplete papilla fill, leading to unfavorable esthetic outcomes^24^. This vulnerability is particularly pronounced in sites with a thin underlying buccal plate, which can compromise implant stability and longevity^25^.

Moreover, the gingival phenotype not only influences the immediate esthetic outcomes but also has implications for long-term implant success. Research indicates that patients with thin gingival phenotypes may experience higher rates of peri-implantitis and mucositis, which can jeopardize implant survival^26,27^. The thin phenotype is often characterized by a delicate and translucent appearance, making it more prone to exposure to the implant surface and subsequent complications^28^. In contrast, thick biotypes tend to maintain the integrity of the soft tissue around implants, thereby providing a more stable biological seal that protects the underlying bone from infection and inflammation^27^. Consequently, understanding the gingival phenotype is crucial for clinicians when planning implant placements, as it can guide decisions regarding soft tissue augmentation procedures and the selection of appropriate surgical techniques to enhance the peri-implant environment^29^. Overall, the gingival phenotype plays a vital role in determining the success and longevity of dental implants, emphasizing the need for personalized treatment approaches based on individual anatomical characteristics.

Thus, the goal of this systematic review was to evaluate the influence of gingival phenotype (thin and thick) on dental implant survival. Thus, the implant success rate for both gingival phenotypes was greater than 91% within a follow-up of up to 5 years. Therefore, it is unclear whether the thickness of the gingival tissue surrounding the implant can directly influence the marginal bone level. The gingival phenotype may be indirectly involved in the survival rate of dental implants, as it can be a risk factor for peri-implantitis, leading to marginal bone loss beyond what is expected. MBL is more related to the type of prosthetic platform of the implant, its 3D position in the dental arch, mainly when placed in the esthetic zone, and the evolution of the severity of peri-implantitis. The treatment approach must be adapted to each phenotype to improve the predictability of functional and esthetic results, especially in the presence of the thin gingiva, which provides lower protection and more frequent maintenance appointments.

### Gingival phenotype

This review found only two types of gingival phenotype classification from the included articles, which are the most found: thin, up to 2 mm thick, and thick, over 2 mm thick. In the literature, two types of periodontal phenotype were also recommended: (1) Flat/Thick and (2) Thin/Scalloped^30,31^. Therefore, a third type of periodontium was recognized and classified as Thick/Scalloped^32,33^. The clinical method to define and categorize the gingival thickness can follow the simplest and most reliable way, using a millimeter periodontal probe at intervals of 0.5 mm or 1.0 mm, known as a transgingival probe^34^. The height of the gingival tissue is also measured with a periodontal probe^35–37^. Other alternatives are also used, such as cone beam computed tomography (CBCT), but this is a more expensive method and requires the involvement of different teams and equipment that are usually present in imaging centers or through the translucency or transparency of the gingival marginal tissue when probed at the bottom of the sulcus, in thin gingival phenotypes, it is possible to see the outline of the probe. This greyish coloration is transparent through the gingiva when a periodontal probe is inserted into the gingival sulcus. In contrast, in thick gingival phenotypes, this diffusion of the greyish color of the probe cannot be observed^38^. However, this method does not accurately determine the thickness of the soft tissue covering the bone. Transgingival was the method of choice for 3 articles in the present review. The lack of uniform, well-defined cross-sectional criteria for evaluating peri-implant soft tissue is a factor that makes it difficult to compare clinical studies in the existing literature.

Every implant undergoes tissue remodeling around it due to the reconstruction of the new STA (former biological width). In dental implants, this can be around 2–3 mm from the gingival margin. If the phenotype is thin, bone resorption around the implant neck compensates for the missing difference in forming STA. Thick biotypes undergo less restructuring compared to thin phenotypes. In the results of this review, Weigl and Strangio’s findings^20^ show no significant differences for 246-thin and 379-thick phenotypes in terms of changes in the peri-implant mucosa. They found a value of less than 0.95 mm for both types, as both did not significantly influence MBL, in line with Guarnieri et al.’s findings^21^, who evaluated 166 implants placed in the upper and lower jaw and also observed that for both phenotypes there were no significant differences; however, the average MBL exceeded the overall average of the articles that made up the results of this research, as well as in the article by Isler et al.^5^, because both authors evaluated patients with peri-implantitis.

### Marginal bone loss (MBL)

Bone loss seems to be more related to the type of implant’s prosthetic connection than to the gingival phenotype. As suggested by Joly et al. ^6^, the selection of tissue- or bone-level implants has no significant differences in relation to natural bone loss. However, the level of implant-abutment connection 2.8 mm above the level of the bone crest may favor less bone loss. Positioning the region where the implant and prosthetic abutment fit together away from the bone crest, it is possible to find a microscopic gap that is passive to the accumulation of microorganisms that could invade the STA. Weigl and Strangio^20^ considered it safe if the implant was placed 1 mm palatally in relation to the cervical emergence profile of the adjacent teeth, with a mesiodistal distance of 1.5 mm and an apical-coronal position of 1 mm apical to the CEJ of the adjacent teeth. The platform switching of the implant, which complements the concept of: “one abutment, one time”, is an asset in preserving the quality of the soft and hard tissues. Constant interventions in the barrier epithelium region resulted in a loss of the horizontal dimension of the bone and increased recession, especially in the middle of the buccal face. The inter-proximal bone level is mainly related to the tooth adjacent to the edentulous area rather than the side in contact with the implant. The proximal papilla can be maintained if the bone peak is preserved during tooth loss^17^.

### Biological complications

The peri-implant phenotype is directly related to gingival morphology and bone structure. Contamination by microorganisms, injuries, iatrogenic factors (excess cement residue, inadequate restoration or abutment seating, implants positioned too far towards the vestibular), and traumatic extractions to the bone structure^8^ can predispose to peri-implant disease and cause gingival damage and morphological changes. As a consequence, it can harm bone tissue to the point of leading to loss of bone structure, changes in the relief of the region surrounded by the affected soft tissue, and compromise the survival of dental implants when they are present in the arch.

Biological complications of implants have been identified as the main reason for dental implant failure. Peri-implantitis is one of the leading causes of failure. The etiology of peri-implant disease is very similar to periodontal disease. Both are caused mainly by the accumulation of bacterial plaque around the natural tooth or implant or microbial invasion of the soft tissues in these sites. If not eliminated, this can lead to local inflammation, which in its initial stages is called peri-implant mucositis and can easily evolve into peri-implantitis, where progressive bone loss can be observed^39^, with PD greater than 3 mm and the presence of BoP and/or suppuration on probing. The absence of horizontal collagen fibers in the peri-implant tissue results in less resistance to probing, which can lead to a local weave with the presence of BoP in sites with peri-implantitis, where it is possible to reach the base of the inflammatory lesion at the level of the alveolar bone crest. In sites with mucositis, the probe’s tip can identify the location of the apical level of the barrier epithelium^10^ and not the level of the bone crest. The barrier epithelium (similar to the junctional epithelium of the periodontium) formed by the soft tissues around implants is the primary protection against invading microorganisms. In contrast to the periodontium, a lower number of fibroblasts is observed in the peri-implant mucosa, which may justify the conclusion of Isler et al. ^5^ that the thin phenotype could be more prone to an increase in the severity of peri-implantitis.

Because a gingival morphology with an adequate keratinized mucosa thickness greater than 2 mm, it can favor the success and maintenance of natural and implanted dental elements when combined with reasonable plaque control, as this ensures greater stability of the peri-implant area and prevention of mucosal recession. However, it does not influence the long-term survival of implants because even if there is no keratinized mucosa or if this tissue is reconstructed using free gingival grafting techniques, it is possible to preserve the implant’s support structures and keep the gingiva free of inflammation and irritation with just adequate routine oral hygiene.

On the other hand, the absence of a keratinized mucosa with a thickness of up to 2 mm around implants is associated with a more significant accumulation of plaque, leading to gingival inflammation, as evidenced by BoP^10^. The lack of keratin makes the mucosa more sensitive to stimuli caused by brushing, which in some cases can result in a sensation of pain, making oral hygiene difficult in some regions of the dental arch. In addition, there is a greater number of leukocytes in the implant barrier epithelium; in the presence of contamination of the peri-implant tissue, it is possible to observe a faster development of the inflammatory process since these cells are responsible for intensifying it^40^. However, in the study by Guarnieri et al.^19^ the incidence of mucositis and peri-implantitis was not associated with the gingival phenotype (*p* > 0.05); then, the gingival phenotype was only a risk factor for peri-implantitis.

The univariate and multivariate statistical analyses applied by Isler et al.^5^ showed that non-adherence to maintenance therapy by patients after implant treatment conferred a significant association with the progression and severity of peri-implantitis in the medium term. In the presence of inflammation, the thin gingival phenotype may be associated with greater marginal recession of the mucosa around implants; bone tissue can suffer rapid loss associated with recession. The thick phenotype, conversely, comprises a dense, fibrous mucosa that provides greater resistance to chemical and mechanical trauma and is susceptible to forming pockets and periodontal granulomas during inflammation^41,42^.

Therefore, the approach to treatment with dental implants must be adapted to each phenotype and be common clinical practice when thinking about the long-term results of implant rehabilitations since thin and thick phenotypes influence the remodeling of hard and soft tissues after tooth extraction or implant procedures, and the final esthetics of the case in the long term^43^, especially in cases where esthetics is highly demanding, such as when one or more anterior teeth are missing. In these cases, a thick phenotype has higher success rates in preserving the interdental papillae than thin phenotypes, which can be prone to papilla loss when the distance from the tip of the papilla to the bone crest is greater than 4 mm.

According to Cosyn et al.^15^, the thin gingival phenotype is a risk factor in cases of immediate implant placement. The level of the papillae in the presence of a thick gingival phenotype can be maintained in cases of immediate implantation when using implants with laser-engraved necks after extraction. However, 2 years later, Guarnieri et al.^19^ observed that the thin and thick soft tissues remained almost stable over time when comparing immediate and delayed implantation cases. This is in line with Weigl and Strangio’s findings^20^, which observed that in 625 implants placed in the anterior maxilla, there were no significant differences in papillae for both phenotypes.

### Clinical strategies suggested

However, surgeons should consider some measures to prevent implant failure. Firstly, it should be made part of the clinical practice to recognize the gingival tissue phenotype and take it into account when planning cases, with the aid of a millimeter periodontal probe and visual inspection of the ridge in terms of its characteristics and region. Using periodontal and surgical strategies minimizes the esthetic and functional consequences that may arise in the short and long term^44^.

In in vivo studies, using 0.12% chlorhexidine digluconate solution and amoxicillin^35^ pre- and post-operatively was correlated to the maintenance of implant success rates above 91%, avoiding infection by pathogenic microorganisms. Atraumatic extractions to preserve bone structure are important to prevent recession and dehiscence defects around implants, especially when thin phenotypes are involved^1^. In these cases of immediate implants^45^, the use of biomaterials such as demineralized and mineralized freeze-dried bone allografts, xenografts (mainly of bovine origin)^46^, and alloplastic biomaterials^47^ barrier membranes, tenting pins, collagen plugs, connective tissue grafts, free gingival grafts, acellular dermal grafts^48^, and buccal flap advancement help to preserve the ridge in most cases^13^.

However, Stiller et al.^18^ stated that it is not known whether the gingival phenotype can influence the treatment of peri-implantitis with soft tissue grafts, suggesting more studies in this area. Otherwise, it is known that it can bring protection benefits at the peri-implant level and more comfort for the patient since it improves the quality of the keratinized mucosa around the implant.

A flapless protocol can also provide a better esthetic result in the short term, although there seems to be no long-term advantage^49^. The professional must manage peri-implant tissues using provisional crowns when the esthetic factor is vital to obtain a harmonious papilla and cervical margin^50^.

The thin gingival phenotype is one of the main risk factors for additional bone loss, as Aguirre-Zorzano et al.^16^ suggested. It is crucial for the long-term success of dental implants that the clinicians know how to preserve them, especially in situations where it is not possible to improve their morphology with grafts, to see the extent to which the various shapes of prosthetic implant platforms, the shape of intermediate prosthetic components and surgical techniques can cause biological trauma, damaging soft and hard tissues.

### Limitations of the study

The study limitations were observed on the lack of statistical analysis, which was due to the high heterogeneity found in the included studies, on the low number of articles included, which could be higher if other databases were included, and on the risk of bias that was predominantly moderate, raising concerns for the data obtained.

## Conclusion

Within the results and limitations found, the gingival phenotype is indirectly related to implant survival rate and clinical parameters, respectively high and non-conclusive. Therefore, a higher risk of peri-implantitis is suggested when a thin phenotype is present. There is a need for more scientific articles attempting to determine more precisely the survival rate of dental implants in relation to the different gingival phenotypes, implementing a solid methodology, such as a randomized controlled trial, with a larger sample size and establishing uniform and cross-sectional criteria in the evaluation of peri-implant soft tissues.

## Supplementary information

PRISMA file

## Data Availability

All the available data was included in the study.
